# Rare Case with Pathogenic Variant in DHX16 Gene Causing Neuromuscular Disease and Oculomotor Anomalies

**DOI:** 10.3390/ijms26062812

**Published:** 2025-03-20

**Authors:** Stefania Kalampokini, Dimitrios G. Goulis, Georgia Pepe, Stavrenia Koukoula, Antonis Frontistis, Maria Moschou, Marianthi Arnaoutoglou, Vasileios Papaliagkas, Vasilios K. Kimiskidis

**Affiliations:** 1 First Department of Neurology, AHEPA University Hospital, Aristotle University of Thessaloniki, Stilponos Kyriakidi 1, 54636 Thessaloniki, Greece; stkalampok@gmail.com (S.K.); antonisfront@gmail.com (A.F.); mar.mos1981@gmail.com (M.M.); marnaoutoglou@yahoo.com (M.A.); kimiskid@auth.gr (V.K.K.); 2 Unit of Reproductive Endocrinology, First Department of Obstetrics-Gynaecology, Aristotle University of Thessaloniki, 56429 Thessaloniki, Greece; dgg@auth.gr; 3 Genekor Medical S.A., 15344 Athens, Greece; gpepe@genekor.com; 4 Vitreoretinal Department, Ophthalmica Eye Institute, 54622 Thessaloniki, Greece; stavreniak@gmail.com; 5 Department of Biomedical Sciences, School of Health Sciences, International Hellenic University, 57400 Thessaloniki, Greece

**Keywords:** *DHX16* mutation, neuromuscular disease, oculoauditory anomalies, DEAH-box helicase

## Abstract

The DEAD/DExD/H-box RNA helicases are a group of RNA-binding proteins involved in the metabolism of mRNAs. They coordinate gene expression programs and play a role in cellular signaling, fate, and survival. We describe a case of a 36-year-old female with neuromuscular disease, sensorineural hearing loss, retinitis pigmentosa, and primary ovarian insufficiency harboring a heterozygous de novo missense pathogenic variant in the DEAH-box helicase 16 (*DHX16*) gene. This is the first case exhibiting a high intellectual level and the highest survival outcome so far. Eight previous cases of *DHX16* disease-causing variant carriers have been described with common features, including muscle weakness with hypotonia, myopathy or peripheral neuropathy, sensorineural hearing loss, abnormal retinal findings, and infantile spasms or epilepsy. Increasing evidence associates RNA-binding proteins, including the DEAD/DExD/H-box helicase family genes, with neuropsychiatric or neurodevelopmental disorders. *DHX16* genetic analysis should be considered early when diagnosing a child or young adult with muscular disease, severe hearing loss, and ocular anomalies.

## 1. Introduction

RNA helicases comprise the largest family of enzymes involved in the metabolism of mRNAs. [1] RNA helicases have several actions, such as mRNA splicing (i.e., the removal of introns from a transcribed pre-mRNA), nuclear mRNA export, the regulation of RNA-RNA and protein–RNA interactions, translation, mRNA decay, cytoplasmic transport, and storage and microRNA-induced gene silencing [1,2]. The most abundant families of RNA helicases are the DEAD box (DDX) and DEAH box (DHX) proteins. [1] The DEAD/DExD/H-box RNA helicases are a group of RNA-binding proteins with ATPase activity, which participate in the unwinding of RNA secondary structures and the remodeling of ribonucleoproteins [1,3]. The *DHX16* gene, mapped on chromosome 6, encodes DEAH-box helicase 16 (DHX16), an ATP-dependent RNA helicase that participates in spliceosome complex B, which is required for mRNA splicing [3]. DEAD/DExD/H-box RNA helicases are widely expressed; nevertheless, some members are specifically expressed in certain tissues [4]. DHX16 is expressed at higher levels in the cerebellum compared to other brain regions [4].

Heterozygous pathogenic variants in *DHX16* have been recently identified in association with a spectrum of clinical phenotypes, including developmental delay, neuromuscular disease, sensorineural hearing loss, and ocular anomalies, which are the so-called “neuromuscular disease and oculoauditory anomalies” or NMOAS (OMIM #618733) [5]. All disease-causing variants in the *DHX16* gene are missense in nature and are found de novo. Mutant DHX16 causes defective spliceosome activity, leading to a catalytically inactive spliceosome complex. This leads to the retention of unspliced pre-mRNA transcripts from several genes in human cell nuclei, deleterious to proper cellular function [6]. Pathogenic variants in other members of the DExD/H-box RNA helicase superfamily cause similar phenotypes with developmental delay and/or intellectual disability [4,7], as well as rare features such as respiratory problems, congenital heart disease, skeletal muscle mitochondrial DNA depletion, and late-onset neurologic decline [8].

We present the case of a 36-year-old female with sensorineural hearing loss, tetraparesis, retinitis pigmentosa, and primary ovarian insufficiency harboring a heterozygous de novo missense pathogenic variant in the *DHX16* gene.

## 2. Case Presentation

**Figure 1:**
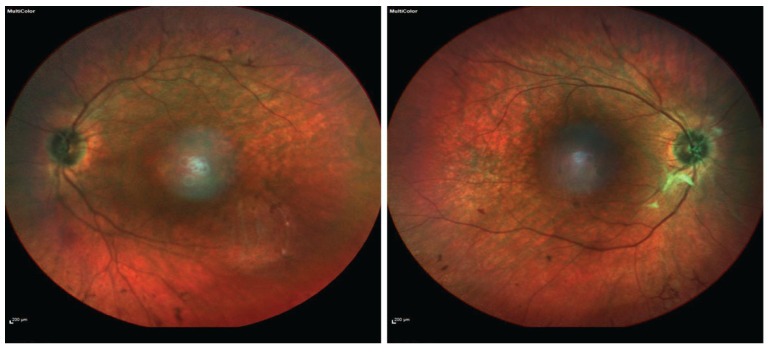
Fundoscopy of the left (**a**) and right (**b**) eye.

**Figure 2:**
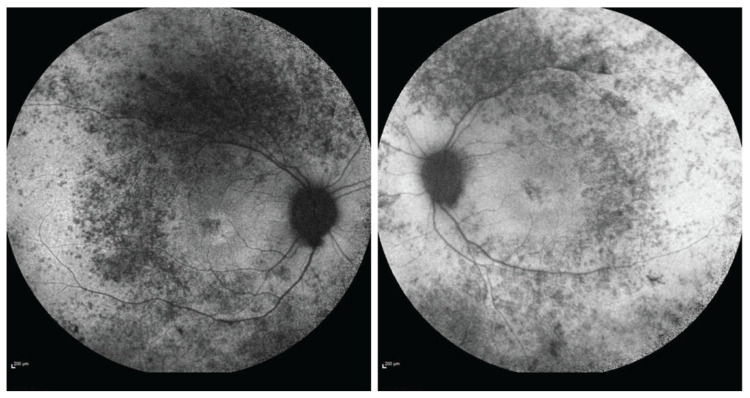
Fundus Autofluorescence of the right (**a**) and left (**b**) eye.

**Figure 3:**
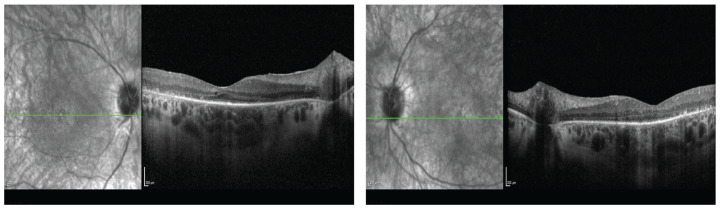
Infrared imaging and optical coherence tomography of the right (**a**) and left (**b**) eye.

A 36-year-old female presented at our Neurology Department for an investigation of long-standing tetraparesis, sensorineural hearing loss, and visual problems. The patient was born full-term and moved normally during pregnancy. There were no problems with sucking or breathing perinatally. She had experienced hearing difficulties since birth that were diagnosed as bilateral sensorineural hearing loss at 13 months of age. The patient first walked at 17 months. At the age of 12, she started to have gait difficulties until the age of 22, when she became a wheelchair user. In parallel, she became progressively weak in her forearms and fingers. Visual symptoms (nyctalopia, photosensitivity, color, and peripheral vision problems) were added at the age of 27. She had reduced visual acuity (6/9 binocular), reduced peripheral vision, and a color vision defect (13/17 Ishihara test), all gradually worsening. Her full-field electroretinogram was severely abnormal (scotopic and photopic) in keeping with rod-cone dystrophy. Her fundoscopy (Figure 1) and imaging (Optical coherence tomography and Fundus Autofluoresence) (Figure 2 and Figure 3) revealed severe retinal dystrophy (retinitis pigmentosa). At the time of presentation, she was on hormone replacement treatment due to primary ovarian insufficiency. Osteoporosis was also present, attributed to immobilization and hypoestrogenemia.

On examination, her intellectual level was normal to high. She had mild facial palsy bilaterally, nasal speech, significant swallowing difficulties, a weak gag and cough reflex, absent deep tendon reflexes, paraplegia, and severe paresis of both upper extremities, with peripheral parts being severely affected. She had winged scapula bilaterally as well as marked lumbar lordosis. Her brain MRI was normal. Lactate was normal, yet the lactate-to-pyruvate ratio was severely increased (≥20). The rest of the serological exams were normal. Nerve conduction studies showed severe axonal sensorimotor peripheral polyneuropathy, whereas electromyography revealed changes compatible with myopathy. Visual-evoked potentials were abnormal bilaterally, showing P100 latency prolongation following the stimulation of the right eye and absence of repeatable waveforms on the left eye. Sensory-evoked potentials revealed a prolongation of N/P37 waveforms. A muscle biopsy was not performed.

Genomic DNA was extracted from the patient sample, and library preparation was carried out using the Nextera DNA Flex kit (Illumina). Whole-genome sequencing (WGS) was performed on the NovaSeq 6000 platform (Illumina, Inc., San Diego, CA, USA) with an average coverage depth of >30X. Variant calling and annotation were performed using the Geneyx platform. Variant filtering was based on population databases (e.g., gnomAD), mutation databases, relevant literature, in silico tools, functional studies, and the patient’s clinical phenotype. Genes associated with the patient’s phenotype were selected according to the OMIM and HPO databases. Variants with a population allele frequency >1%, unless previously reported as pathogenic or likely pathogenic, and variants classified as benign or likely benign were excluded from the report, as they are unlikely to impact disease risk or medical management. Point mutations and copy number variations (CNVs) unrelated to the patient’s referral reason were also not reported. Variants of uncertain significance (VUS) in genes potentially associated with the phenotype were included, especially those predicted to impact protein function by computational tools (e.g., REVEL, MetaLR).

Genetic testing through whole-genome analysis and next-generation sequencing revealed a heterozygous missense pathogenic variant in the *DHX16* gene NM_003587.5:c.2032G>A, p (Glu678Lys) on chromosome 6 in exon 13 in the helicase C-terminal domain. The sequence change replaces glutamic acid with lysine at codon 678. This variant is not present in population databases such as gnomAD, Clin/Var, ExAC, and 1000G. The impact of this variant on the protein’s structure and function was assessed using the REVEL score, which ranges from 0 to 1 for a missense variance, with higher values indicating a higher probability of pathogenicity [9]. The revel score of our finding p.(Glu678Lys) was 0.93, indicating that the variant may impact the structure or function of the protein. In our analysis, the variant was classified as likely pathogenic based on the ACMG/AMP criteria PM2, PM5, PM6, and PP3 [10]. The PM5 criterion was applied because the variant c.2033A>G, p.(Glu678Gly) at the same amino acid position (p.Glu678) has been previously reported as a likely pathogenic variant. It was not identified in the parents of the patient (i.e., de novo). Another heterozygous variant, NM_001384474.1:c.4480C>T, p.(Arg1494*), was found in exon 29 in the *LOXHD1* gene on chromosome 18. The variant in the LOXHD1 gene was classified as likely pathogenic based on the ACMG/AMP criteria PVS1, PS4, PM2 [10].

The patient received a nutrition supplement containing myoinositol, a-lipoic acid, L-carnitine fumarate, L-arginine, N-acetylcystein, B5, and Q10 (2500 mg/day, 1070 mg/day, 625 mg/day, 625 mg/day, 625 mg/day, 7.5 mg/day, and 312.5 mg/day, respectively) in light of the findings of severely increased lactate to pyruvate ratio in order to enhance mitochondrial function. During the one-year follow-up, her clinical picture was stable.

## 3. Discussion

**Figure 4:**
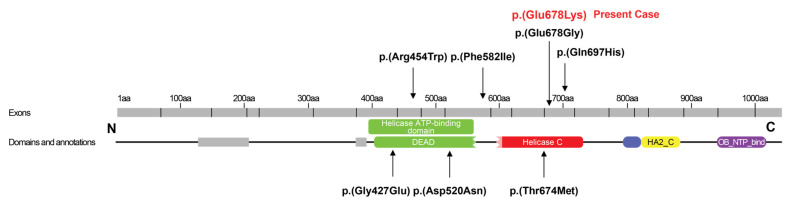
Schematic representation of the DHX16 protein with its known functional domains and previously published pathogenic DHX16 variants (the novel variant is shown by the red color). DEAD: DEAD-like helicases superfamily domain containing the ATP-binding region; helicase C: helicase superfamily C-terminal domain; HA2_C: helicase-associated domain (HA2), with as-yet-undetermined specific function; and OB_NTP_bind: oligonucleotide/oligosaccharide-binding (OB)-fold, central to helicase activity modulation.

Eight previous cases with patients carrying a *DHX16* mutation have been reported (Figure 4, Table 1). Paine et al. first reported a cohort of four infants with de novo missense variants in *DHX16*; the three presented with combinations of developmental delay, neuromuscular disease, seizures, ocular or retinal abnormalities, and sensorineural hearing loss, while one died shortly after birth [4]. Park et al. reported the case of an individual with congenital myopathy and de novo heterozygous mutation p.(Thr674Met) as part of a Korean whole-exome sequencing study in patients with neurodevelopmental disorders [11]. Archana et al. reported an 18-month-old toddler with infantile spasms, hypotonia, sensorineural hearing loss, and optic and retinal abnormalities carrying a heterozygous missense mutation c.1445G > A p.(Arg482His) [12]. Drackley et al. reported a case of a 16-year-old patient with neuromuscular disease, hearing loss, retinal degeneration, and previously unreported phenotypic features including mitochondrial deficiency and primary ovarian insufficiency, carrying a c.2033A>G p.(Glu678Gly) mutation [5] at the same amino acid position at the C-terminal as in the present case, suggesting that variants that disrupt this residue are likely to be disease-causing. Hautakangas et al. reported an infant with a heterozygous de novo p.(Arg454Trp) mutation in the ATP-binding region of the DHX16 protein, with developmental delay, hypotonia and, later on, spasticity, dysmorphic features, oculoauditory anomalies, elevated creatine kinase levels, and additional intellectual disability [13].

The present case is the ninth reported case of a patient harboring a heterozygous *DHX16* missense de novo disease-causing variant, causing neuromuscular disease and oculoauditory anomalies with additional phenotypic features of primary ovarian insufficiency and retinitis pigmentosa, which have rarely been previously reported [5,13]. Our case was 36 years of age, which is significantly older compared to most other cases, highlighting the remarkable heterogeneity of this rare entity in terms of the survival outcome. She also exhibited a high intellectual level, as opposed to previously reported cases carrying pathogenic variants in other members of the DExD/H-box RNA helicase superfamily, causing developmental and/or intellectual disability [4,7,8].

Although no single feature is characteristic for *DHX16* pathogenic variant carriers, severe sensorineural hearing loss (6/9 cases), muscle weakness with hypotonia, myopathy or peripheral neuropathy (5/9 cases), abnormal retinal findings (5/9 patients) and epilepsy/infantile spasms (3/9 cases) are common features. Poor growth, small size, and/or dysmorphic features with epicanthal folds were reported in two patients [4]. Only one patient developed a movement disorder with ataxic gait [4]. Except for one case with corpus callosum agenesia and subependymal heterotopia [4], no case showed brain structural anomalies. The presence of atypical features, such as primary ovarian insufficiency and cystic kidneys, makes clinical diagnosis challenging. The fact that DExD/H-box helicases are involved in cellular signaling, fate, and survival [1] might explain how *DHX16* pathogenic variants result in phenotypic diversity, i.e., diverse features from several systems. Developmental and/or intellectual delay, seizures, and dysmorphic features were also common traits in individuals with pathogenic variant in other helicase family genes, such as *DHX34*, *DHX37*, and *DHX54* [4]. The differential expression of helicase family genes in the affected tissues may also explain the different phenotypes [4].

The *DHX16* phenotypic spectrum resembles mitochondrial disorders with multisystemic involvement, sensorineural hearing loss, retinal involvement (especially retinitis pigmentosa), epileptic activity, and myopathy/elevated creatine kinase levels. Notably, one patient exhibited reduced activity of cytochrome C oxidase, citrate synthase, and complex IV, as well as a mitochondrial depletion syndrome in mitochondrial DNA analysis, pointing to a mitochondrial deficiency [5]. Furthermore, a severely increased lactate-to-pyruvate ratio was found in our case, which is observed in mitochondrial respiratory chain defects. Few RNA helicases are located in mitochondria, affecting their function and cell energy metabolism; one example is *DHX32* [14]. However, it cannot be denied that mtDNA depletion is a secondary finding of muscle atrophy [15]. Our patient received a nutritional supplement containing Q10 (312.5 mg/d), as in the case of mitochondrial disorders, and did not show further deterioration at the one-year follow-up. Another important differential diagnosis of the DHX16 phenotypic spectrum is a rare, potentially treatable, metabolic disorder, caused by defective riboflavin transporters encoded by SLC52A2 and SLC52A3 genes [16,17]. This syndrome (OMIM #211530), previously known as the Brown–Vialetto–Van Laere or Fazio Londe syndrome, is also characterized by sensorineural hearing loss, facial and limb weakness, sensorimotor axonal neuropathy, feeding, and respiratory difficulties; however, it can be significantly improved by high-dose riboflavin supplementation [18,19].

The location of amino acid substitution in the present case is on a helicase C-terminal domain, which is a region important for the function of DHX16 protein. The pathogenic variant in one case [5] was found in the same amino acid location with the substitution of glutamine acid by glycine p.(Glu678Gly), while, in our case, glutamine was substituted by lysine. The two cases share several common features, such as sensorineural hearing loss, hypotonia, myopathy, neuropathy, and primary ovarian insufficiency, with the latter reported only in those two cases. At present, it seems unclear whether the location of pathogenic variant in the C-terminal of the protein or in the ATP-binding area affects the severity of the phenotype. Nevertheless, the reported cases are very few. Thus, the issue of whether distinct locations of pathogenic variants within the protein functional domains have differential impacts on the protein function and, therefore, explain the different phenotypes remains to be elucidated in the future.

Increasing evidence associates RNA-binding proteins, including the DEAD/DExD/H-box helicase family genes, with neuropsychiatric or neurodevelopmental disorders [4]. RNA helicases function as molecular drivers of the progression of mRNA substrates either to a productive mRNA pool leading to protein synthesis or to unproductive mRNA that is stored or degraded [1]. They can function as fate switchers by driving specific mRNA transcripts from one pool to another at any step along this process [1]. RNA helicases have pleiotropic functions at different steps of gene expression, monitoring the direction of the flow of genetic information [1]. Thus, RNA helicases have a prominent role in orchestrating gene expression programs in response to extracellular cues and during cell differentiation [1].

Furthermore, our patient had a heterozygous c.4480C>T, p.(Arg1494*) variant in the exon 29 in the *LOXHD1* gene located on chromosome 18, inherited with an autosomal recessive form and associated with non-syndromic hearing loss [18]. It encodes a highly conserved protein, the Lipoxygenase homology domain 1 (LOXHD1), expressed in hair cells of the cochlea and vestibule [19]. Biallelic pathogenic variant (either homozygous or compound heterozygous) in *LOXHD1* are the cause of DFNB77 (DEAFNESS, AUTOSOMAL RECESSIVE 77, OMIM #613079), which is a recently described, rare, highly heterogeneous condition causing progressive and severe-to-profound non-syndromic hearing loss [18]. The onset age varies from neonate to adulthood; however, most patients develop hearing loss before 5 years of age [19]. The heterozygous combination of one missense and one splicing or biallelic missense variant, common in Europe, is more likely to lead to a race-specific milder phenotype than other combinations [18]. To what extent this heterozygous mutation contributed to our patient’s phenotype is unclear, as she had congenital deafness.

This report adds to our knowledge of the association of rare pathogenic missense variants of the *DHX16* gene with neuromuscular disease and oculomotor disorders. *DHX16* genetic analysis should be considered early when diagnosing a child or young adult with severe hearing loss, muscular disease, and ocular anomalies. Diagnosing such complicated phenotypes is challenging due to wide genetic heterogeneity and rare occurrence, while whole-exome sequencing facilitates the diagnosis. A future challenge will be identifying which particular helicases contribute to the production and usage of specific mRNAs in given tissues. Even more challenging is the development of molecules targeting the enzymatic activity of RNA helicases or modulating the ATPase activity of RNA helicases, which may be useful for cell-type reprogramming.

## Figures and Tables

**Table 1:** Case reports of patients with *DHX16* gene pathogenic variants.

Authors, Year	Pathogenic Variant	DHX16 Domain	Inheritance	Gender	Age of Onset	Age of Diagnosis	Neurological Symptoms	Other Features	MRI Brain	First Symptom	Outcome
Paine et al., 2019 [4] individual 6	c.1744 T > A p.(Phe582Ile) heterozygous	C-terminal	De novo	Female	10 weeks	n/a	Hypotonia, poor head control, poor visual tracking, infantile spasms	Sensorineural hearing loss, chorioretinal lacunae, depigmentation around optic nerve	Corpus callosum agenesis, subependymal heterotopia	Infantile spasms	n/a
Paine et al., 2019 [4] individual 7	c.2091G > T p.(Gln697His) heterozygous	C-terminal	De novo	Female	Birth	Infancy	n/a	Small length, short limbs, dysmorphic facial features (bilateral epicanthal folds, simple auricles), enlarged cystic kidneys	n/a	Decreased fetal heart tones, reduced birth length, dysmorphic features	Death 16 days after birth
Paine et al., 2019 [4] individual 8	c.1280G > A p.(Gly427Glu) heterozygous	ATP-binding area	De novo	Male	Birth	Infancy	Sensorimotor neuropathy, horizontal nystagmus	Sensorineural hearing loss, bilateral equinovarus, feeding difficulties, respiratory distress, flexion contractures	Normal	Severe hypotonia, skeletal abnormalities	Death at the age of 4 months
Paine et al., 2019 [4] individual 9	c.2021C > T p.(Thr674Met) heterozygous	C-terminal	De novo	Male	10 months	Infancy	Epilepsy, developmental delay, myopathy, peripheral neuropathy, hypertrophic calves, ataxic gait, unable to walk	Sensorineural hearing loss, retinopathy, complete vision loss, contractures	Normal	Febrile seizure	At age 34 wheelchair-bound, blind, deaf
Archana et al., 2022 [12]	c.1445G > A p.(Arg482His) heterozygous	ATP-binding area	Unknown	Male	18 months	Toddler (18 months)	Hypotonia, nystagmus, developmental delay, infantile spasms	Sensorineural hearing loss, blindness, retinal pigmentary spotting	Normal	Sensorineural deafness at 4 months of age	n/a
Park et al., 2022 [11]	c.2021C > T p.(Thr674Met) heterozygous	C-terminal	De Novo	n/a	Birth	n/a	Myopathy	n/a	n/a	n/a	n/a
Hautakangas et al. 2023 [13]	c.1360C>T p.(Arg454Trp) heterozygous additional homozygous missense variant c.1378C>G p.(Arg460Gly) in CHRNB2 gene	ATP-binding area	De Novo	Female	Infancy (3 months)	n/a	Hypotonia, lack of eye contact, elevated CPK, constant nystagmus, spasticity	Sensorineural hearing loss, dysmorphic features (epicanthal folds, single palmar crease, sandal gaps), poor weight gain, pale fundi with pigmentation and depigmentation, failure to thrive	Normal	Dysmorphic features	Death at 4 years
Drackley et al., 2024 [5]	c.2033A > G p.(Glu678Gly) heterozygous	C-terminal	De novo	Female	Birth	15 years	Hypotonia, motor delay, sensorimotor axonal neuropathy, myopathy, ADHD	Bilateral sensorineural hearing loss, retinitis pigmentosa, premature adrenarche, primary ovarian insufficiency	n/a	Bilateral sensorineural hearing loss	n/a
Present case	c.2032G>A, p.(Glu678Lys) heterozygous	C-terminal	De novo	Female	Birth	36 years	Facial palsy, swallowing difficulties, nasal speech, myopathy, flaccid tetraparesis	Sensorineural deafness, retinitis pigmentosa, primary ovarian deficiency	Normal	Sensorineural hearing loss	Deaf, wheelchair-bound

## Data Availability

The data presented in this study are available upon request from the corresponding author.
